# Effect of Time on the Properties of Bio-Nanocomposite Films Based on Chitosan with Bio-Based Plasticizer Reinforced with Nanofiber Cellulose

**DOI:** 10.3390/ijms241713205

**Published:** 2023-08-25

**Authors:** Weronika Janik, Michał Nowotarski, Kerstin Ledniowska, Natalia Biernat, Abdullah, Divine Yufetar Shyntum, Katarzyna Krukiewicz, Roman Turczyn, Klaudiusz Gołombek, Gabriela Dudek

**Affiliations:** 1 Łukasiewicz Research Network—Institute of Heavy Organic Synthesis “Blachownia”, 47-225 Kędzierzyn-Koźle, Poland; weronika.janik@icso.lukasiewicz.gov.pl (W.J.); kerstin.ledniowska@icso.lukasiewicz.gov.pl (K.L.); natalia.biernat@icso.lukasiewicz.gov.pl (N.B.); 2 PhD School, Department of Physical Chemistry and Technology of Polymers, Silesian University of Technology, 44-100 Gliwice, Poland; abdullah.abdullah@polsl.pl; 3 Department of Physical Chemistry and Technology of Polymers, Faculty of Chemistry, Silesian University of Technology, 44-100 Gliwice, Poland; michnow566@student.polsl.pl (M.N.); katarzyna.krukiewicz@polsl.pl (K.K.); roman.turczyn@polsl.pl (R.T.); 4 Biotechnology Centre, Silesian University of Technology, 44-100 Gliwice, Poland; divine.yufetar.shyntum@polsl.pl; 5 Centre for Organic and Nanohybrid Electronics, Silesian University of Technology, 44-100 Gliwice, Poland; 6 Materials Research Laboratory, Faculty of Mechanical Engineering, Silesian University of Technology, 44-100 Gliwice, Poland; klaudiusz.golombek@polsl.pl

**Keywords:** chitosan, nanofiber cellulose, aging, bio-based plasticizer

## Abstract

The deterioration of the performance of polysaccharide-based films over time, particularly their hydrophilicity and mechanical properties, is one of the main problems limiting their applications in the packaging industry. In the present study, we proposed to improve the performance of chitosan-based films through the use of: (1) nanocellulose as an additive to reduce their hydrophilic nature; (2) bio-based plasticizer to improve their mechanical properties; and (3) chestnut extract as an antimicrobial agent. To evaluate their stability over time, the properties of as-formed films (mechanical, hydrophilic, barrier and antibacterial) were studied immediately after preparation and after 7, 14 and 30 days. In addition, the morphological properties of the films were characterized by scanning electron microscopy, their structure by FTIR, their transparency by UV-Vis and their thermal properties by TGA. The films showed a hydrophobic character (contact angle above 100°), barrier properties to oxygen and carbon dioxide and strong antibacterial activity against Gram-negative (*E. coli*) and Gram-positive (*S. aureus*) bacteria. Moreover, the use of nanofillers did not deteriorate the elongation at breaks or the thermal properties of the films, but their addition reduced the transparency. In addition, the results showed that the greatest change in film properties occurred within the first 7 days after sample preparation, after which the properties were found to stabilize.

## 1. Introduction

The research interest in the development of innovative and environmentally friendly biocomposites based on biodegradable polymers and fillers to improve material strength properties has continued to grow rapidly over the last few decades. Among the various fillers for biopolymers, nanofillers are among the most popular, not only for their reinforcing properties but also for providing additional advantages, such as barrier properties or antibacterial activity [1]. Nanofillers play an important role in the design of biopolymer-based materials for various applications. Their main task is to improve the mechanical properties of the material, which has already been confirmed in numerous studies [2,3,4,5]. The most commonly used biopolymer nanofillers for food packaging films are cellulose, starch, chitin and chitosan [6]. Some of the nanofillers, such as nanochitosan, improve not only the mechanical properties of the film but also its antimicrobial properties [7].

Among the previously mentioned nanofillers, nanocellulose (NC) is the most popular. It is a biodegradable and renewable nanofiller, obtained as a result of the decomposition of cellulose fibers [8]. The high interest in it is mainly due to its wide availability—cellulose is the main polysaccharide accumulating in plant biomass [2]. It is estimated that plants produce about 75 billion tons of cellulose per year, making it a highly available material [9]. Three types of nanocellulose are used in applications that improve material mechanical properties: cellulose nanofibers, cellulose nanocrystals and bacterial nanocellulose. The first two are isolated from plants and the last from bacteria [2]. Cellulose fibers, with or without chemical pretreatment, are mechanically fragmented in water into nanocellulose. The obtained products are distinguished depending on their morphology into nanofibrils and nanocrystals [8].

The use of NC is becoming also increasingly popular due to its properties, such as high strength, high extensibility, non-toxicity and biodegradability [10]. The incorporation of NC into the polymer matrix allows for the formation of a material with improved performance without compromising its biodegradability [11]. The effect of introducing NC into bio-based materials has been widely studied [4,7,12,13]. Attempts to obtain NC-reinforced chitosan nanocomposites suitable for use in the food industry, e.g., as active packaging films, have been described [2,11,14]. It has been observed that the addition of nanocellulose leads to the improvement of the mechanical properties, thermal stability, hydrophobicity and oxygen barrier of the film. Moreover, NC-loaded films showed bactericidal activity against Gram-positive and Gram-negative bacteria and fungicidal activity against *Candida albicans* [11]. In addition, chitosan films with NC reduce total volatile basic nitrogen (TVB-N), which is considered an indicator of food freshness under refrigeration conditions [11].

Despite ongoing research into combining chitosan with NC for use as bioactive packaging films, the impact of time on such systems remains unclear [11]. This information is crucial from the perspective of applying these materials for food packaging. The issue of the instability of chitosan–glycerol compositions has already been addressed by us in our previous study [15], in which we proved that replacing glycerol with esters of propylene glycol and acetic acid resulted in a more stable material over time. Therefore, in the present study, the esters of propylene glycol and acetic acid were also used for plasticization and were tested not only immediately after receiving them but also after 7, 14 and 30 days. The maximum time was determined based on our previous studies conducted on films based on chitosan and sodium alginate [15,16]. To obtain bioactive films, chestnut extract was used. To further improve the properties of chitosan, a biocomposite was obtained using NC as a filler. Mechanical properties, hydrophilic properties, oxygen, carbon dioxide and water vapor permeability and antibacterial activity were determined for the obtained sample immediately after receiving the samples and after 7, 14 and 30 days. To evaluate the morphology, structure, transparency and thermal properties of the obtained samples, scanning electron microscopy, Fourier transform infrared spectroscopy, ultraviolet-visible spectroscopy and thermal gravimetry were used, respectively.

## 2. Results and Discussion

### 2.1. Mechanical Properties

**Figure 1:**
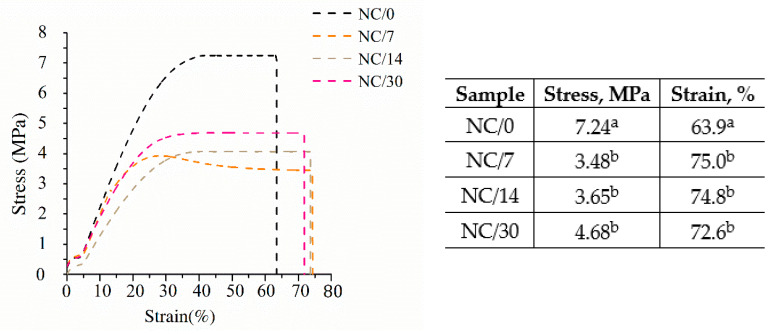
Stress vs. strain curves with exact values of bio-nanocomposite films based on chitosan; different lowercase letters indicate significantly different values at *p* < 0.05 using Tukey’s multiple range test.

The mechanical performance of polymeric materials is one of the most important properties determining their applicability. Tensile strength (TS) and elongation at break (EB) for bio-nanocomposite films based on chitosan were measured immediately after the film preparation and after storage at room temperature for 7, 14 and 30 days to determine the effects of time on the mechanical properties. The characteristic stress–strain curves of the bio-nanocomposite films are illustrated in Figure 1. The strongest effect of time on mechanical performance can be observed within the first 7 days after sample preparation. The TS value drops during this time from 7 MPa (NC/0) to 3.48 MPa (NC/7), and then for the subsequent days, the values are statistically equal. After 14 days of sample preparation, the TS value was 3.65 MPa (NC/14), and after 30 days, it increased slightly to 4.68 MPa (NC/30). The presented results show that the first 7 days have the greatest effect on the deterioration of TS. In contrast, the EB results show a slightly different relationship, as the elongation increased slightly over time. Indeed, newly prepared film showed an EB of 63% (NC/0), while after 7 days, this value increased to 75% (NC/7), and then for the subsequent days, the values were statistically equal. For NC/14, the EB was 74%, and for NC/30, it was 72%. This outcome is primarily related to the deteriorating properties of TS, which mostly cause the EB to increase [17,18]. Comparing the results of our previous studies [15] conducted without the use of NC, it can be seen that the presence of nanofibers significantly affects the increase in TS. Chitosan films prepared with the same plasticizer but without NC showed a TS of 6 MPa, while the use of nanofibers increased this value to 7 MPa (NC/0), and after 30 days, it dropped to 4.5 MPa (NC/30). In contrast, EB values remained at the same level, i.e., about 70%. The same relationship was observed by other researchers [3,5,7,11], who also reported a significant effect of nanofiller on TS and not on EB. For example, Costa et al. [11] added cellulose nanocrystals to a chitosan film and observed that, as the amount of NC increased, TS increased, while EB remained unchanged. Nevertheless, this relationship is not confirmed in every case, as in the study described by Jannatyh et al. [3], the addition of cellulose nanocrystals to the chitosan film resulted in an increase in TS and a simultaneous decrease in EB. This outcome may be related to poor dispersion of the nanofiller, incompatible structures or agglomeration of the nanoparticles [19]. Also worth mentioning is the effect of the chestnut extract itself used in the present samples. It has been shown that the addition of chestnut extract causes an increase in TS, but a decrease in EB [20,21]. This finding was confirmed, among others, by the study of Kõrge et al. [21], who observed a significant increase in TS and decrease in EB for chitosan-based films after the addition of chestnut extract.

### 2.2. Hydrophobic Properties

**Figure 2:**
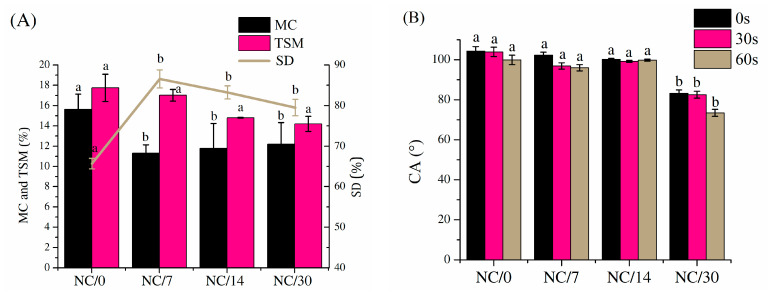
Moisture content (MC), total soluble matter (TSM) and swelling degree (SD) (**A**) and dynamic contact angle (CA) (**B**) for bio-nanocomposite films based on chitosan; different lowercase letters indicate significantly different values at *p* < 0.05 using Tukey’s multiple range test.

**Figure 3:**
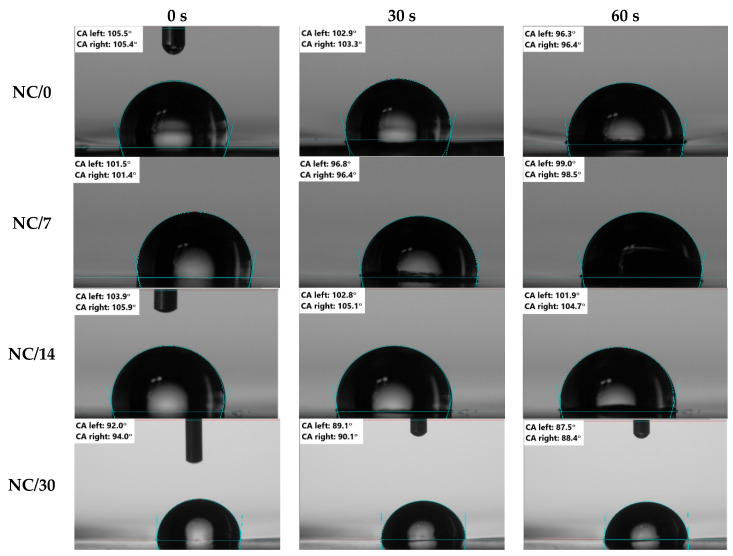
Dynamic contact angle images of bio-nanocomposite films based on chitosan.

The hydrophobic properties of polymeric materials are as important as the mechanical properties for the determination of their applicability. Moisture content (MC, Figure 2A), total soluble matter (TSM, Figure 2A), swelling degree (SD, Figure 2A) and dynamic contact angle (CA, Figure 2B and Figure 3) for chitosan-based bio-nanocomposite films were measured immediately after film preparation and after storing the films for 7, 14 and 30 days to determine the effects of time on the hydrophobic properties. The results showed that all of these values decreased slightly over time. For MC, the initial value dropped from 15% (NC/0) to about 11% for the other samples (i.e., NC/7, NC/14 and NC/30). TSM values also dropped slightly, from about 18% for NC/0 to about 14% for NC/30. In the case of SD, the values increased after 7 days from 50% to 85% (for NC/0 and NC/7, respectively) and then dropped slightly to 81% and 78% (for NC/14 and NC/30, respectively). Most of these results also showed that the stabilization of the material occurred after 7 days, as the largest changes were recorded during this time, and the other values were statistically the same A similar conclusion was observed in the mechanical properties discussed earlier, which decreased over 7 days related to the decrease in moisture content and thus to the formation of a less flexible structure through a reduction in plasticization of the amorphous areas of the polymer network [22,23].

In contrast, the wetting angle results shown in Figure 2B and Figure 3 indicate that the sample tested immediately after preparation showed a hydrophobic character (100° at 0 s and about 98° at 30 s, NC/0) and this value, despite the decreasing MC value over time, remained unchanged until 14 days (100°; NC/14). However, tests performed on day 30 showed a decrease in the wetting angle value to about 82° (NC/30), demonstrating that the samples became hydrophilic over time. It is also worth mentioning the effects of NC and chestnut extract itself on these properties. Referring to the results obtained in previous studies [15], MC, TSM or SD for pure film without NC is practically the same as for film with nanofiller. In contrast, the CA results showed that the use of NC shifted the nature of the film decisively from hydrophilic to hydrophobic (from 70° to 105° for films without NC and with NC, respectively). The improvement of hydrophobic properties was also observed by Lavrič et al. [2], in which the addition of cellulose nanocrystals to the chitosan film led to an increase in the contact angle from 75° to 108°. This relationship was also confirmed by Mao et al. [4], who showed that, after adding cellulose nanocrystals to the chitosan film, CA increased. Moreover, the value continuously increased by increasing the NC content. On the other hand, in the case of the addition of chestnut extract, Bajić et al. [20] and Kõrge et al. [21] observed that, with an increase in its amount (0.5–1%), the hydrophilicity decreased. This outcome was attributed to the presence of a large number of hydrolysable tannins in the chestnut extract; this component has many interaction sites that crosslink the polymer chain, so they tend to saturate the hydrogen bonds in chitosan.

### 2.3. Gas Permeability

The barrier properties of polymer films play an important role in the food packaging industry. The film is designed to delay the transfer of molecules between food and the environment to preserve food quality. Measuring properties, such as the permeability of oxygen, carbon dioxide or water vapor, makes it possible to estimate the shelf life of a product. The most commonly studied properties of biodegradable films are water vapor permeability and oxygen permeability because the proper amount of water keeps products fresh and crispy, while oxygen spoils food through oxidation reactions but is necessary for respiration of fresh vegetables and fruits [24].

**Figure 4:**
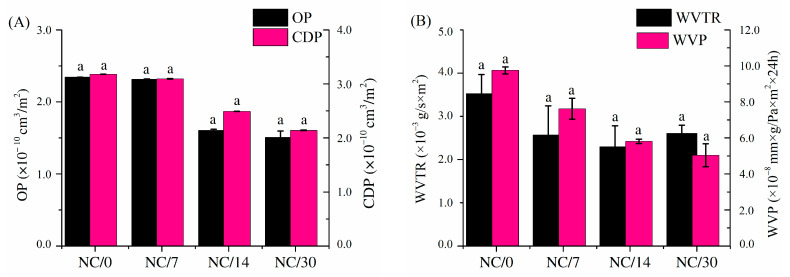
Oxygen (OP) and carbon dioxide permeability (CDP) (**A**) and water vapor transmission rate (WVTR) and water vapor permeability (WVP) (**B**) for bio-nanocomposite films based on chitosan; different lowercase letters indicate significantly different values at *p* < 0.05 using Tukey’s multiple range test.

Oxygen and carbon dioxide permeability (Figure 4A) and water vapor transmission rate and water vapor permeability (Figure 4B) for chitosan-based bio-nanocomposite films were measured immediately after film preparation and after storing the films for 7, 14 and 30 days to determine the effects of time on barrier properties. The obtained results of both OP and CDP for the tested films were relatively low (at a level of 10^−10^ cm^3^/m^2^) compared to commercially available polylactide film or low-density polyethylene (PE-LD) [25] and slightly decreased over time. The OP for NC/0 was about 2.5 × 10^−10^ cm^3^/m^2^, and after 30 days, this value decreased to about 1.5 × 10^−10^ cm^3^/m^2^ (NC/30). In contrast, the CDP for NC/0 was about 3 × 10^−10^ cm^3^/m^2^, and after 30 days, the value dropped to about 2 × 10^−10^ cm^3^/m^2^ (NC/30). Despite the notable decline in values, none of these results were statistically different from each other. The OP of chitosan films is influenced by the concentration of plasticizer, as well as storage time. Leceta et al. [26] reported that OP values increased with increasing glycerol content. In addition, Butler et al. [27] reported that OP increased not only with the plasticizer concentration but also after film storage time. A similar decreasing relationship over time was noted for WVP and WVTR. The obtained values were relatively low and close to each other, and after 30 days, they reached a value of about 5 × 10^−8^ mmxg/Paxm^2^ × 24 h (NC/30). As in the previously described studies, i.e., mechanical and hydrophobic properties, the greatest changes for WVP and WVTR were observed after 7 days of storage, whereas for OP and CDP, a decrease in these values was observed after 14 days of storage. Nevertheless, also in this case, the results were not statistically different from each other. It is worth mentioning that slightly higher WVP values may be related to higher relative humidity in the environment [28,29,30] because chitosan has amino and hydroxyl groups that interact with water molecules through hydrogen bonds without modifying the chemical structure [22,31]. Adsorption of water molecules allows for a more flexible structure by plasticizing the amorphous regions of the polymer network and promoting internal rearrangement, consequently facilitating gas transport through the polymer matrix [22,23]. Moreover, WVP is closely related to the MC of the material: as WVP increases, MC also increases [18]. In our study, MC decreased over time, which was also observed for WVP, OP and CDP, due to the evaporation of water from the polymer matrix under the storage conditions. As the water content of the material decreases, its elasticity decreases, making the structure more compact and resistant to water and gas permeability [32].

Comparing the barrier properties (OP, CDP and WVP) obtained for plasticized chitosan film without nanofillers in our previous study [15] to the results obtained with the addition of NC, it can be seen that the addition of nanofiller slightly increased the values of OP, CDP and WVP, but these values were still relatively low values, and the obtained barrier results were satisfying because the barrier properties of the obtained bio-nanocomposite films based on chitosan films exceeded or were almost equal to the commercial food packaging films currently used, such as those based on PLA or PE-LD [25,26]. The introduction of NC deteriorated the barrier properties of the film because it facilitated the diffusion of the gas molecules through the film structure. NC in the form of fibers creates channels that facilitate the passage of oxygen, carbon dioxide or water vapor molecules through the film [33]. For pure chitosan film, Kerch et al. [34] reported that WVP increases at room temperature during storage (30 days). In contrast, Khan et al. [35] studied the effect of NC concentration on the WVP of chitosan films. They reported that WVP values decreased with increasing NC concentrations. In their case, crystalline NC was used and not the fiber form, which had a significant impact on the results.

### 2.4. Morphology

**Figure 5:**
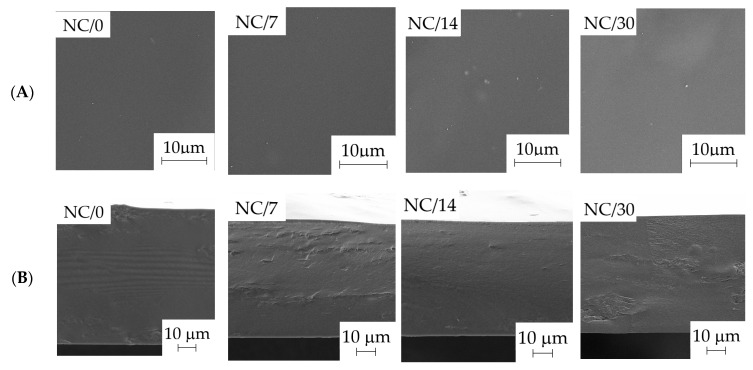
SEM images of the bio-nanocomposite films based on chitosan: surface (**A**) and cross-section (**B**).

To study the structure of the obtained bio-nanocomposite films based on chitosan, surface and cross-sectional images (Figure 5A,B, respectively) were taken to evaluate the homogeneity of the obtained materials. SEM microphotographs show the homogeneity of the NC-loaded film, as confirmed by both its surface and cross-sectional images. The surface of the chitosan films was found to be smooth, which indicated good film homogenization of chitosan and NC in aqueous medium. Considering the possibility of agglomeration of the used nanofiller in the film, no significant inhomogeneities that could indicate a large agglomeration of the filler were noted. The films showed a homogeneous and dense structure, indicating proper NC dispersion in the chitosan matrix.

### 2.5. Fourier Transform Infrared Spectroscopy

**Figure 6:**
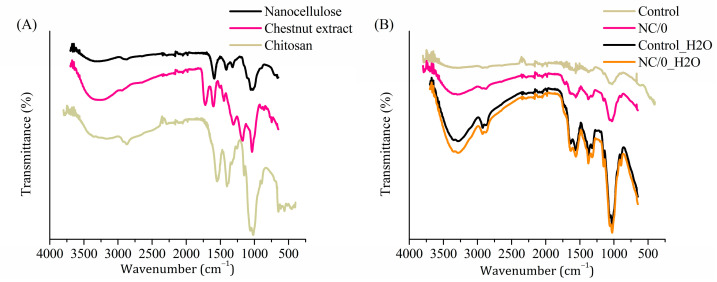
FTIR spectra of the NC, chestnut extract and chitosan powders (**A**), of the obtained films before and after immersion in water (**B**), where control indicates plasticized chitosan film with chestnut extract, and H2O indicates after immersion in water.

FTIR analysis was carried out to evaluate the structural interactions among chitosan, NC and chestnut extract and the structural changes of the film after exposure to water for 24 h. Figure 6A shows the spectra of NC, chestnut extract and chitosan powders, and Figure 6B showed the plasticized chitosan film with chestnut extract (Control), with chestnut extract and nanocellulose (NC/0) and after their immersion in water for 24 h (Control_H2O and NC/0_H2O, respectively). The FTIR spectrum of NC powder (Figure 6A) showed several typical bands, namely the very broad bands located in the region of 3200–3500 cm^−1^, corresponding to stretching vibrations of the O–H groups of cellulose, a C–H stretch band at 2900 cm^−1^ and absorption bands of β-glycoside bonds at 1591 and 1057 cm^−1^ [11,36,37,38]. The powder spectrum of chestnut extract (Figure 6A) also showed its characteristic bands in the region of 3100–3500 cm^−1^, corresponding to the O–H stretching vibrations derived from different chemical environments, characteristic of polyphenolic extracts [39,40]. In the region of 2800–2985 cm^−1^, the C–H stretching vibrations, derived from carbohydrates and sugars, could be observed and, between 1661 and 1769 cm^−1^, the C=O stretching vibrations of esters of hydrolysable tannins. In the region of 1422–1620 cm^−1^ the C=C-C aromatic bonds [39] and, in the region from 1123 to 1380 cm^−1^ and from 959 to 1082 cm^−1^, the bands for C–O were observed, respectively [39,40]. Structural analysis of chitosan powder (Figure 6A) also showed characteristic bands, i.e., between 3100 and 3500 cm^−1^, the overlapping broad band from both O–H and N–H stretching vibrations and, at 2920–2850 cm^−1^, the symmetric and asymmetric modes of C–H stretching vibrations at methylene and methyl carbon [41,42]. Since the chitosan grade is 90% DD, the characteristic peaks representing the I amide band (C=O stretching in nondeacetylated amide) were only weakly visible in the slope of the free amine N–H bending and C–N stretching located at 1555 cm^−1^. The weak resolved broad peak with a maximum at 1400 cm^−1^ is attributed to the combined C–H bending in -CH and -CH_2_, as well as -CH_3_ symmetrical deformations and -CH_2_ wagging [26]. The absorption band at 1038 cm^−1^ could be attributed to the C–O stretching and asymmetric stretching of the C-O-C bridge in the saccharides ring. The signal at 893 cm^−1^ corresponds to the out of the plane C–H bending of the saccharides ring [26].

Figure 6B shows the plasticized chitosan film with chestnut extract (Control) and the film with chestnut extract and nanocellulose (NC/0), as well as after their immersion in water for 24 h (Control_H2O and NC/0_H2O, respectively). Comparing the spectra before and after soaking in water, a significant increase in band intensity was observed for both Control_H2O and NC/0_H2O. After soaking the spectra, all of the characteristic features of the bio-composite were retained. More intensive stretching vibrations of the O–H groups in the region of 3100–3500 cm^−1^ were noted, which can be attributed to the absorbed residual water. The observed increase in the intensity and sharpness of the peaks characteristic for chitosan indicated that 24 h of soaking in water led to its swelling and backbone relaxation, and the residual water likely partially softened the composite, ensuring its better adherence to the UATR crystal surface. No evident release of bio-composite components, such as plasticizer or fillers, was observed.

### 2.6. Optical Properties

**Figure 7:**
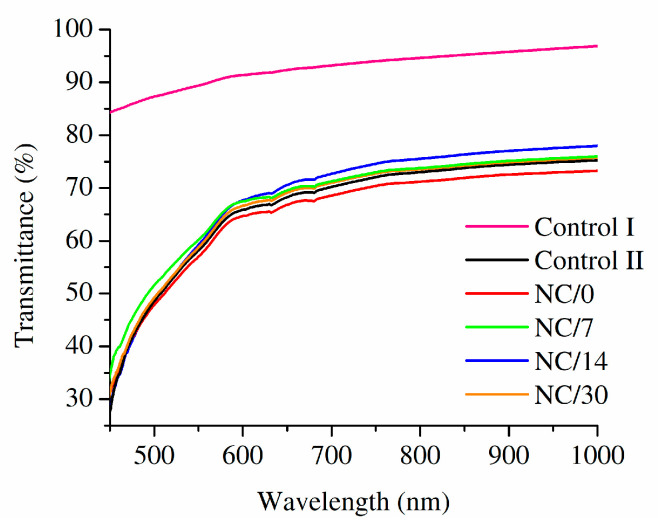
UV-Vis spectra of the obtained NC-loaded films based on chitosan, as well as chitosan films without (Control I) and with chestnut extract (Control II).

An important property of food packaging materials is their transparency since it allows consumers to evaluate food freshness and general appearance by visual inspection before purchasing [43]. Based on the UV-Vis spectra of the obtained bio-nanocomposite films based on chitosan, as well as chitosan films with and without a chestnut extract (Figure 7), it can be observed that the presence of chestnut extract decreased the transmittance of films, even without the presence of NC fibers. While the transmittance of a pristine chitosan film was noted between 84% (at 450 nm) and 97% (at 1000 nm), it dropped to 28–75% for chestnut extract-containing chitosan film. The presence of NC decreased transmittance further, but this feature was found to increase with time, as the process of aging occurred. The observed discoloration should be associated with the limited stability of chestnut extract and its partial degradation [44].

### 2.7. Antimicrobial Activity

**Figure 8:**
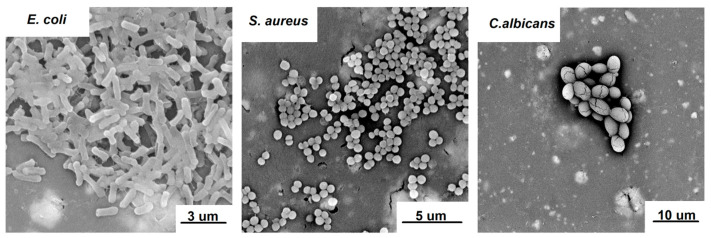
SEM images of *E. coli*, *S. aureus* and *C. albicans* inoculated overnight on the surface of NC/0 films.

Antimicrobial activity of NC-loaded films was assessed toward *Escherichia coli*, *Staphylococcus aureus* and *Candida albicans*, which represent model foodborne pathogens that are Gram-negative bacteria, Gram-positive bacteria and fungi, respectively [45,46,47]. Their morphology after 24 h of incubation on the surface of NC/0 is presented in Figure 8. Observed phenotypes indicated that the pathogens entered the multiplication stage of growth that occurred after their attachment to the surface. Therefore, 24 h was found to be an appropriate timepoint to investigate the effect of the material on the viability of pathogens, eliminating the risk of false-positive results based on the presence of pathogens entering the death phase [48].

**Figure 9:**
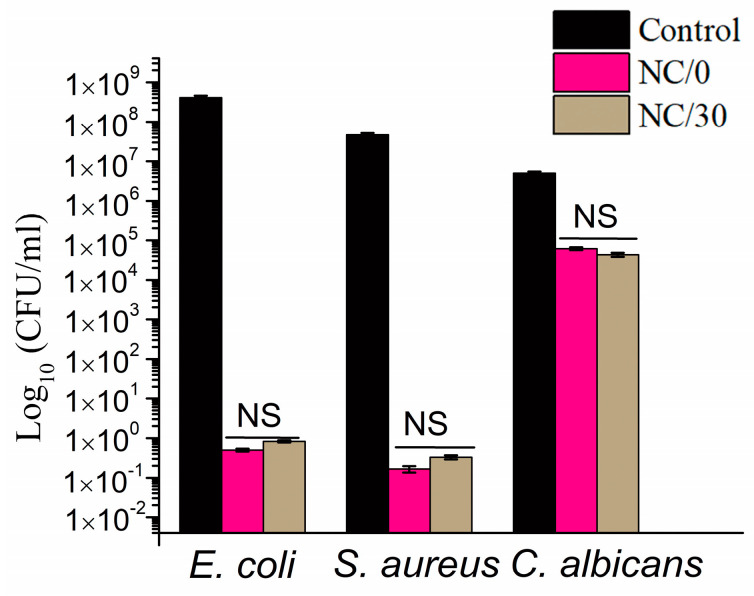
Antimicrobial activity of bio-nanocomposite films based on chitosan against *E. coli*, *S. aureus* and *C. albicans*, where Control—chitosan-based film without NC; NS = no significant difference relative to the controls. Comparisons among groups were performed by one-way ANOVA, followed by Bonferroni’s multiple comparison post-hoc test. Statistical significance was considered at *p* < 0.05.

The results of the antimicrobial tests (Figure 9) showed that the chitosan-based bio-nanocomposite films obtained in this study caused a strong reduction in the growth of both model Gram-negative (*E. coli*) and Gram-positive (*S. aureus*) bacteria and slight fungicidal activity against *C. albicans*. In particular, a 6–7 log reduction in the growth of *E. coli* and *S. aureus* was observed, while only a 1–2 log reduction was observed for *C. albicans*. In addition, the study showed that the chitosan-based bio-nanocomposite films were stable over time and retained their antibacterial activity against each pathogen. It is important to mention that the source of the antimicrobial activity of the studied films was the presence of both chestnut extract, which is a rich source of polyphenols, such as phenolic acids and tannins [49], and NC, which also exhibits antibacterial activity [50]. The antimicrobial activity of chestnut extract was proven in previous studies [21,25,51], in which its antimicrobial properties were demonstrated against model Gram-positive bacteria (*Staphylococcus epidermidis ATCC12228*), Gram-negative bacteria (*Escherichia coli ATCC25922*) and yeast (*Candida albicans ATCC18804*). Kõrge et al. [51] also confirmed the antibacterial activity of chestnut extract against *Escherichia coli K12* and *Bacillus subtilis DSM 402*. Dehnad et al. [14] prepared films of glycerol-plasticized chitosan and nanocellulose, studying their effects on inhibitory activity against both Gram-positive (*S. aureus*) and Gram-negative bacteria (*E. coli* and *S. enteritidis*). The results proved that, for all of the aforementioned bacteria, there was a decrease in their growth. In turn, Costa et al. [11] investigated the potential of chitosan/cellulose nanocrystal films for use as active pads in meat packaging to extend shelf life and preserve properties over time. Several concentrations of nanocellulose (5, 10, 25 and 50 wt.%) were used, and the films were produced by solvent casting. The study showed that the films obtained in this way exhibited antibacterial activity against Gram-positive and Gram-negative bacteria and slight fungicidal activity against *C. albicans.*

### 2.8. Thermal Analysis

**Figure 10:**
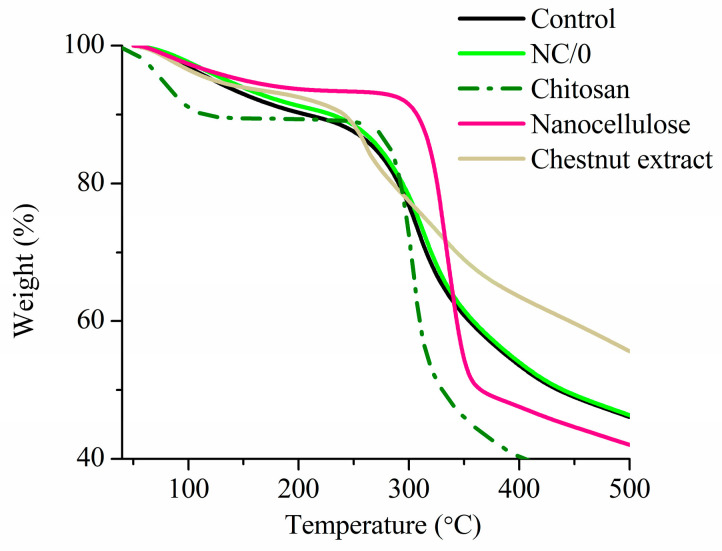
TG curves of the chitosan-based films, neat chitosan, nanocellulose and chestnut extract powder, where Control indicates plasticized chitosan film with chestnut extract.

Thermal degradation of the obtained bio-nanocomposite films based on chitosan (NC/0) was analyzed and compared to the film without NC (Control) and to the used neat chitosan, nanocellulose and chestnut extract (Figure 10). This analysis helped to determine the temperature at which the material is stable and the change in mass as a function of temperature change. Because of the law of NC content, the results indicated that the film with nanocellulose (NC/0) exhibited similar thermal degradation behavior to the film without nanocellulose (Control). They underwent an initial slight weight loss at around 100 °C, followed by a rapid weight loss between 250 and 400 °C. The first stage is associated with the evaporation of water and residual acetic acid present in the polymer matrix [52], while the second thermal event is attributed to the complex decomposition of chitosan [53]. Concerning chitosan, the main stage of thermal degradation begins at 300 °C, which is associated with the greatest weight loss (≈60%). This result can be attributed to degradation of the polysaccharide and deacetylation of chitosan [54]. In the case of nanocellulose, two steps were also noted, namely dehydration and degradation. The complete release of absorbed water took place at around 100 °C, followed by sharp decomposition noted at around 320–350 °C. In the case of the nanocellulose, the degradation occurred at ca. 50–70 °C lower than that typical for cellulose. The same relationship was also observed in previous work [55], indicating that nanocellulose should not be processed at temperatures higher than 200 °C. In the case of chestnut extract, initial weight loss was observed due to water evaporation, and the moisture was completely evaporated at about 150°, followed by the second phase, described as multistep complex thermal degradation (starting around 220 °C) [56].

## 3. Materials and Methods

### 3.1. Materials

Chitosan 0.03–0.1 Pa × s (MW = 250 kg/mol, DD ≥ 90%) was purchased from Sigma-Aldrich (Steinheim, Germany). Acetic acid was purchased from Avantor (Gliwice, Poland) (99.5–99.9%), and Farmatan chestnut extract (≥76% tannins) was provided by Tanin Sevnica (Sevnica, Slovenia). Propylene glycol, cyclohexene (pure p.a.) and sodium hydrogen carbonate (pure p.a.) were purchased from Chempur (Piekary Śląskie, Poland). Methanesulfonic acid (>99.0%) was provided by TCI (Zwijndrecht, Belgium). Nanofibrillated cellulose (10–20 nm wide, 2–3 µm length) was purchased from Nanografi Nano Technology (Ankara, Türkiye).

### 3.2. Preparation of the Bio-Based Plasticizer

Plasticizer mixtures were prepared as described in our previous study [15,57]. In short, propylene glycol was esterified with acetic acid for 24 h at 80 °C using methanesulfonic acid as a catalyst. Cyclohexane was used to remove water as a byproduct of the reaction. The esterification product was purified using a saturated solution of sodium hydrogen carbonate, distilled water and cyclohexane.

### 3.3. Preparation of the Bio-Nanocomposite Films

Bio-nanocomposite films based on chitosan with a bio-based plasticizer reinforced with nanofiber cellulose were prepared by a casting method as described in our previous study [15]. Chitosan (2%, *w*/*v*) was dissolved in 1.0% (*v*/*v*) acetic acid aqueous solution with 30% (*w*/*w*) bio-based plasticizer based on the mass of chitosan by stirring with a magnetic stirrer at 800 rpm at room temperature for 24 h. Chestnut extract (0.75% (*w*/*v*)) was added, and the solution was homogenized for 5 min at 6000 rpm. Then, 0.5% (*w*/*v*) nanocellulose was added and homogenized for 1 min at 800 rpm. These solutions were left overnight to let the air bubbles disappear. Finally, bio-nanocomposite chitosan solutions were cast over Petri dishes (46 g per 12 cm × 12 cm dish) and dried at room temperature. In this way, bio-nanocomposite films with a thickness of about 94 µm were obtained and tested directly (sample NC/0) and after 7, 14, and 30 days (sample NC/7, NC/14, and NC/30, respectively) of storage at 23 °C ± 2 °C and 50 ± 5% relative humidity with access to light and air.

### 3.4. Mechanical Properties

Tensile tests were performed at room temperature using an Instron 4466 testing machine. The specimens were cut in rectangular shapes 80 mm in length and 20 mm in width. The samples were conditioned before testing for 24 h at 23 ± 2 °C and 50 ± 2% RH and were stretched at a crosshead speed of 5 mm/min. All tests were carried out on a minimum of five samples, and the final results were calculated as an average. The entire procedure was repeated for films stored for 7, 14 and 30 days.

### 3.5. Hydrophilic Properties

The hydrophilicity of the films was analyzed by moisture content—MC, swelling degree—SD, total soluble matter—TSM and water contact angle—CA. MC, SD and TSM were analyzed using a three-step gravimetric method; i.e., film samples with surface area of 1 cm^2^ were weighed (M_1_), dried at 100 °C for 24 h and weighed again (M_2_).

(1) $$\;\mathrm{MC}\left(\%\right)=\frac{\left(M_{1}-M_{2}\right)}{M_{1}}\times 100$$

The samples were then placed in 30 mL of distilled water, left at room temperature for 24 h and weighed again (M_3_).

(2) $$\;\mathrm{SD}\left(\%\right)=\frac{\left(M_{3}-M_{2}\right)}{M_{2}}\times 100$$

In the final step, the samples were dried at 100 °C for 24 h and weighed (M_4_). Measurements were repeated five times, and the average value was calculated. TSM values were calculated using the following formula:

(3) $$\;\mathrm{TSM}\left(\%\right)=\frac{\left(M_{2}-M_{4}\right)}{M_{2}}\times 100$$

The dynamic water contact angle of the film surface was measured using a semi-automatic goniometer for dynamic contact angle and contour analysis systems (OCA15EC, DataPhysic, Filderstadt, Germany). A droplet of 5 μL of deionized water was applied on the film surface according to the device’s instructions. The measurement was taken immediately upon contact with the surface, after 30 s and after 60 s. The dynamic water contact angle was determined based on the average of the three measurements. The entire procedure was repeated for films stored for 7, 14 and 30 days.

### 3.6. Gas Permeability

Oxygen and carbon dioxide permeability through the bio-nanocomposite films were determined using an isobaric apparatus [15]. The samples for oxygen and carbon dioxide barrier testing, in the form of discs (60 mm^2^), were degassed for 24 h and conditioned with the appropriate gas in the apparatus prior to testing for 2 h. Then, the diffusion chamber was sealed, and compressed oxygen (class 5.0) or carbon dioxide (technical gas) was supplied at a controlled flow rate to keep the pressure constant. The permeation coefficient was determined as follows:

(4) $$P=\frac{V\times l}{S\times \Delta p}$$

where V is the volumetric flow (mol∙s^−1^), l is the sample thickness (m), S is the sample area (m^2^), and Δp is the pressure difference on both sides of the sample (Pa). The entire procedure was repeated for films stored for 7, 14 and 30 days.

Water vapor transmission rate (WVTR) and water vapor permeability (WVP) were determined according to the methodology proposed by Aguirre-Loredo et al. [32]. The samples in the form of discs (24.64 mm^2^), were mounted on a glass container with silica gel in its interior (~0% relative humidity, RH) and sealed with liquid paraffin. After the paraffin solidified, the cup was weighed to calculate the initial weight. The covered glass container was then placed in a desiccator containing a supersaturated saline solution of BaCl_2_ (90% RH), generating a water-vapor differential pressure of 2854.23 Pa. The glass container was weighed seven times at 60-min intervals. The determinations were made in triplicate. The entire procedure was repeated for films stored for 7, 14 and 30 days. The WVTR and WVP values were determined as follows:

(5) $$\mathrm{WVTR}=\frac{\Delta m}{\Delta \mathrm{tA}}$$

(6) WVP=WVTR×LΔp

where ∆m/∆t is the moisture weight gain in time (g/s), A is the exposed surface area of the film (m^2^), L is the thickness of the film (mm), and ∆p is the difference in partial pressure (Pa).

### 3.7. Morphology

The morphology of the bio-nanocomposite films was examined by a scanning electron microscope (ZEISS Supra 35) at 10 kV of accelerating voltage, equipped with an energy dispersive X-ray detector (EDS) (Thermo Scientific™ EDX UltraDry).

### 3.8. Fourier Transform Infrared Spectroscopy

Fourier transform infrared (FTIR) spectra of the composite films were recorded in the range of 3700–650 cm^−1^ with resolution of 2 cm^−1^ using a Spectrum Two spectrometer equipped with a diamond UATR accessory (Perkin Elmer). For each spectrum, 16 scans were taken. These analyses were performed in duplicate at room temperature. In addition, the films were also tested after soaking for 24 h in water. For this purpose, the films were cut into small disks and stirred in water at room temperature for 24 h. The following films were dried at 80°C, and spectrum scans were performed according to the above procedure.

### 3.9. Transparency

The transparency of NC-loaded films was measured with the use of UV-Vis spectrophotometry (Hewlett Packard 8453 UV/Vis Diode Array Spectrophotometer) in the wavelength range from 450 nm to 1000 nm. UV-Vis spectra of NC-loaded chitosan films were compared with UV-Vis spectra of pristine chitosan films with and without chestnut extract.

### 3.10. Antimicrobial Activity

The antimicrobial activity of bio-nanocomposite films based on chitosan was determined against *Escherichia coli* ATCC25922, *Staphylococcus aureus* ATCC23235 and *Candida albicans* ATCC18804. They all represent foodborne pathogens [45,46,47], and numerous studies have used these pathogens to validate the antimicrobial activity of various materials, including food packaging materials [58,59]. Samples in the form of discs (10 mm in diameter) were placed in 12-well plates containing 500 µL of M9 minimal medium supplemented with glucose as the sole carbon source. Thereafter, 20 µL of the targeted bacterial culture, normalized to 10^4^ CFU/mL, was inoculated into each well and incubated overnight at 37 °C with shaking at 250 rpm. Overnight cultures were serially diluted in double-distilled autoclaved water and plated on LB agar to determine CFU/mL of recovered targeted bacteria. In control experiments, bacteria were inoculated in M9 media in the absence of NC. Surviving bacteria were quantified by serial dilution and plating on LB agar. All experiments were performed in triplicate and repeated three times.

To visualize and analyze the morphology of pathogens, a scanning electron microscope (Phenom ProX, Thermo Fisher Scientific, Waltham, MA, USA) at 15 kV of accelerating voltage was used. The materials were first fixed with 3% glutaraldehyde (Fisher BioReagents, Waltham, MA, USA) for 24 h and dehydrated by immersing the samples in solutions of ethanol (Acros Organics) with increasing concentrations (30%, 50%, 70%, 80%, 90%, 95%, 99.8%) and then dried for 24 h at 50 °C. Subsequently, the samples were sputter-coated with a gold layer (20 min, 20 mA; Q150R Quorum Technologies, Lewes, UK).

### 3.11. Thermal Analysis

Thermogravimetric analysis (TGA) was carried out using a TGA 8000 thermogravimetric analyzer (PerkinElmer Inc., Waltham, MA, USA). The samples (ca. 10 mg) were heated in an open ceramic sample pan in the temperature range of 50–1000 °C, with the heating rate β = 20 °C/min in a dynamic (20 mL/min) nitrogen atmosphere. The thermographs were collected and analyzed using Pyris^TM^ 11 software (Waltham, MA, USA).

### 3.12. Statistical Analysis

Experimental data in Section 3.1, Section 3.2 and Section 3.3 were analyzed for statistical significance by analysis of variance (ANOVA) and Tukey’s multiple range test with a *p* < 0.05 significance level. Data were evaluated by OriginPro software, version 8.5.0 (OriginLab, Northampton, MA, USA), whereas for the experimental data in Section 3.6, the analysis was performed by one-way ANOVA, followed by Bonferroni’s multiple comparison post-hoc test. Statistical significance was considered at *p* < 0.05

## 4. Conclusions

Nanocomposite films have been made from chitosan, bio-based plasticizer and nanocellulose. The properties of these films were evaluated immediately after preparation and after 7, 14 and 30 days to determine changes over time. Interestingly, the greatest changes in film properties occurred within the first 7 days, after which the properties were found to stabilize. This outcome shows that significant changes occur in the first few days after sample preparation, such as a decrease in MC, which affects the other properties of the obtained films. Immediately after preparation, the tested films showed tensile stress at around 7 MPa, which dropped to 4.5 MPa after 7 days and oscillated around 4 MPa after 14 and 30 days. Elongation at break, on the other hand, increased slightly over time, rising from 60% for the initial sample to about 75% for the remaining ones. In this case, the greatest change also occurred within the first 7 days. The introduction of nanocellulose into the films mainly increased their hydrophobic character (the contact angle was about 105°). Based on the results, the use of nanocellulose to enhance the hydrophobic character of the films may be an interesting method to obtain films with better hydrophobic properties. Due to the presence of chestnut extract and NC, all films exhibited strong antibacterial activity against Gram-negative (*E. coli*) and Gram-positive (*S. aureus*) bacteria and slight fungicidal activity against *C. albicans*, which were maintained over time. The use of nanofillers did not affect the thermal properties of the film, but their addition reduced the transparency. In addition, the results show that testing bio-films not only immediately after testing but also over time can provide more meaningful results. The results presented in this article show not only the possibility of obtaining a more hydrophobic (due to the nanocellulose), more antibacterial (due to the synergistic effect of the chestnut extract, nanocellulose and bioplasticizer) and more durable (due to the bioplasticizer and nanocellulose) chitosan-based film than those without the above-mentioned components, but they also prove that, during the first 7 days, the film’s properties can significantly deteriorate. In our case especially, a significant decrease in tensile strength was noted. Considering that the mechanical properties are among the most problematic properties of chitosan-based films, it is worth noting that films tested directly can provide misleading results. Nevertheless, properties such as antibacterial activity and barrier properties did not deteriorate over time, allowing us to conclude that the films obtained in the present study can successfully serve as active food packaging that extends the life of the product. However, to confirm their applicability to food, further research is needed, including mainly studies of migration performed in terms of the bioplasticizer, as well as the chestnut extract and nanocellulose.

## Data Availability

Not applicable.
